# Morphological changes and lateralization of the thalamic nuclei in children with growth hormone deficiency

**DOI:** 10.3389/fendo.2025.1678738

**Published:** 2026-03-04

**Authors:** Joo Young Lee, Sung-min Lim, Jung Hwan Oh, Hyuna Kim, Gang Yi Lee, Bong Gun Lee, Jeong-Kyu Hoh, Seung Yang, Hyun Ju Lee

**Affiliations:** 1 Department of Translational Medicine, Hanyang University Graduate School of Biomedical Science and Engineering, Seoul, Republic of Korea; 2 Department of Pediatrics, Hanyang University Hospital, Seoul, Republic of Korea; 3 College of Medicine, Hanyang University, Seoul, Republic of Korea; 4 Department of Orthopedic Surgery, Hanyang University College of Medicine, Seoul, Republic of Korea; 5 Department of Obstetrics and Gynecology, Hanyang University College of Medicine, Seoul, Republic of Korea; 6 Department of Obstetrics and Gynecology, Hanyang University Hospital, Seoul, Republic of Korea; 7 Department of Pediatrics, Hanyang University College of Medicine, Seoul, Republic of Korea; 8 Division of Neonatology and Development Medicine, Seoul Hanyang University Hospital, Seoul, Republic of Korea

**Keywords:** growth hormone deficiency, idiopathic short stature, insulin-like growth factor-1, insulin-like growth factor binding protein-3, lateralization, pituitary gland, thalamus

## Abstract

**Introduction:**

Childhood growth hormone deficiency (GHD) is an endocrine disorder characterized by reduced secretion of growth hormone (GH), leading to impaired linear growth and delayed developmental milestones. Recent studies suggest that GHD is associated with cognitive, socio-emotional, and behavioral impairments, potentially via altered neurodevelopment, with neuroimaging studies revealing changes in brain morphology and broader GH-related effects on the central nervous system. The thalamus, a major subcortical relay integrating sensory, motor and cognitive information, has received limited attention in neuroimaging studies of children with GHD. This study aimed to investigate morphological alterations and characterize lateralization patterns of thalamic nuclei in children with GHD.

**Methods:**

Fifteen children diagnosed with GHD and fifteen age- and sex-matched children with idiopathic short stature (ISS) were recruited. The pituitary gland was segmented using ITK-SNAP software. Thalamic nuclei were delineated and parcellated into ten regions using Bayesian MRI methods and a probabilistic atlas. Lateralization indices were calculated as: ((Left − Right)/(Left + Right)) × 100. Group comparisons and correlation analyses were conducted with age and sex as covariates. All volumes were normalized to total intracranial volume (tICV).

**Results:**

Children with GHD exhibited a significantly smaller pituitary gland volume compared to those with ISS, even after adjustment for age, sex, and tICV. In children with GHD, the anteroventral (AV) thalamic nucleus showed increased volume, and the ventral anterior (VA) nucleus exhibited significantly greater leftward asymmetry compared to ISS. Moreover, there was a significant positive correlation between the lateralization index (LI) of the AV nucleus and serum IGF-1 levels (*p* = 0.022) and between the LI of the VA nucleus and serum IGF-1 levels (*p* = 0.022). Similarly, the LI of the AV nucleus was significantly positively correlated with serum IGFBP-3 levels (*p* = 0.022), and there was also a significant correlation between the LI of the VA nucleus and serum IGFBP-3 levels (*p* = 0.033).

**Conclusion:**

The observed leftward lateralization in the anterior thalamic nuclei, together with associations with serum IGF-1 and IGFBP-3 levels, suggests that thalamic lateralization reflects a neurodevelopmental adaptation to disrupted GH signaling. These findings suggest that GH/IGF activity shapes subcortical development in a dose-dependent manner and reveal structural adaptations in hormone-sensitive regions to early endocrine disruption.

## 1 Introduction

Growth hormone deficiency (GHD) is a pediatric endocrine disorder characterized by insufficient secretion of growth hormone (GH) from the anterior pituitary gland, leading to impaired linear growth and delayed growth milestones. Furthermore, GHD impairs not only somatic growth but also brain development through alterations in the GH/Insulin-like growth factor-1 (IGF-1) axis. Short stature affects approximately 2.5% of the pediatric population, with idiopathic short stature (ISS) accounting for 60–80% of the children and GHD for approximately 10% (1, 2). Children with ISS have normal growth hormone secretion with near-normal growth velocity and bone age, whereas those with GHD present with significantly reduced GH levels, decreased growth velocity, and delayed bone age. Although GH is traditionally associated with promoting physical growth, it also plays a crucial role in the development and function of the central nervous system (CNS) (3). Previous studies have reported that patients with GHD may experience various cognitive and neurological difficulties, including social-emotional/behavioral problems, and attention-deficit hyperactivity disorders (4–6). These alterations may influence critical neurodevelopmental processes, particularly in the brain, potentially leading to behavioral changes during childhood and adolescence. GHD has been implicated in processes, such as neurogenesis, synaptic plasticity, and glial function, mediated in part through the GH/IGF axis (7–10). These findings suggest that GHD may contribute not only to impaired somatic development but also to structural and functional alterations in the brain, particularly in domains associated with memory, learning, and other cognitive functions during critical periods of development.

Magnetic resonance imaging (MRI) is a valuable non-invasive tool for assessing pituitary gland morphology in pediatric patients with a suspected endocrine disorder. As the primary source of GH secretion, the pituitary gland has been a central focus in anatomical studies of GHD. Previous studies suggest that children with GHD may exhibit reduced pituitary height or volume compared to age-matched controls, implying a potential relationship between morphometric alterations in the pituitary gland and GH secretory status (11–13). However, most existing studies have relied on conventional two-dimensional (2D) MRI slices, which may fail to capture the full volumetric complexity of the gland. By comparison, high-resolution three-dimensional (3D) MRI allows for direct volumetric segmentation, offering enhanced anatomical precision. Despite these advantages, the use of semi-automated or fully-automated segmentation techniques in 3D MRI remains limited in children with GHD, underscoring the need for more advanced and standardized volumetric approaches.

While previous studies have primarily focused on pituitary morphology, recent evidence indicates that GHD is associated with widespread cortical and subcortical alterations, including structures critical for cognitive and motor functions. Zhang et al. reported significant morphological changes in the cerebral cortex of children with GHD, partially modulated by circulating GH and IGF-1 levels (14). The GH/IGF-1 signaling pathway influences the densely innervated cerebral cortex (15), and consistent with this, global structural analyses have revealed widespread reductions in cortical development in affected children (14, 16). Moreover, neuroimaging studies have increasingly demonstrated both structural and functional brain alterations in children with GHD, particularly in the subcortex, basal ganglia and limbic system (4, 17, 18). GH exerts its effects, directly or indirectly, through IGF-1, a critical mediator of neuronal growth, dendritic arborization, and synaptogenesis during brain development (9, 19). IGF-1 receptors are most densely expressed in the hippocampus, amygdala, thalamus, and prefrontal cortex, implicating these regions in GH/IGF-1–mediated neurodevelopmental processes (9, 10, 20). Disruption of this signaling pathway may underly the structural changes observed in subcortical regions. For instance, Webb et al. reported that volumetric reductions in the corpus callosum, hippocampus, and globus pallidum in patients with GHD were significantly correlated with IGF-1 (4). GH receptor concentrations are particularly high in the thalamus and several other regions of the brain. Although the thalamus is a major subcortical structure, it has received limited attention in neuroimaging studies of children with GHD and ISS. Given its critical role in relaying and integrating sensory, motor, and cognitive information, further investigation into thalamic morphometric characteristics in patients with GHD is warranted. Therefore, the aims of the study were to evaluate pituitary gland morphology using semi-automated and manual segmentation approaches and to investigate thalamic morphometric alterations using advanced neuroimaging methods in children with GHD and ISS, thereby elucidating the impact of GHD on thalamic structure and providing new insights into neurodevelopmental processes.

## 2 Methods

### 2.1 Participants

This study was part of a cross-sectional study of children who visited the Department of Pediatric Endocrinology, Hanyang University College Medicine. Written informed consent was obtained from the guardians of all participants prior to enrollment, and the study was conducted in accordance with the ethical principles of the Declaration of Helsinki, revised in 2013. A total of 30 children aged 4 to 6 years were recruited between July 2016 and January 2023, including 15 children diagnosed with GHD and 15 with ISS, matched for age and sex. Children presenting with short stature underwent GH stimulation testing to differentiate between GHD and ISS. The inclusion criteria for children with GHD were as follows: (I) GHD was diagnosed according to the guidelines for GHD (Cook and Rose, 2012); (II) peak serum GH level less than 10 ng/mL on at least two GH stimulation tests; (III) no other clinically significant neurological or psychiatric disorders, nor adrenocorticotropic hormone deficiency, hypoglycemia, thyroid-related, genetic, or metabolic diseases; (IV) in cases without radiological abnormalities on MRI; and (V) no prior history of GH therapy. Children with ISS met the same diagnostic criteria but exhibited a peak serum GH level exceeding 10 ng/mL in response to stimulation with at least one of the two agents—arginine or levodopa (L-dopa). Participants were excluded if they met any of the following criteria: (I) growth or developmental delay due to other causes, such as small for gestational age, intrauterine growth restriction, or prematurity; (II) a history of GH replacement therapy; or (III) brain lesions detected on MRI or a history of psychiatric or neurological disorders.

### 2.2 Clinical assessment and GH stimulation test

Clinical data were obtained from medical records and included age, sex, height, weight, body mass index, and serum IGF-1 and IGF-binding protein 3 (IGFBP-3) levels. Serum IGF-1 and IGFBP-3 levels were further converted to standard deviation scores (SDS) using age- and sex-specific reference intervals established for Korean children (21). The IGF-1/IGFBP-3 molar ratio was calculated according to the formula as previously described (22):


$$\frac{IGF-1}{IGFBP-3}\text{molar ratio}=\frac{IGF-1\left(\frac{ng}{mL}\right)*0.13}{IGFBP-1\left(\frac{ng}{mL}\right)*0.035}$$

GH stimulation testing was performed to evaluate GHD and remains the gold standard for the clinical differentiation of short stature in children with either GHD or ISS (23). A clinical diagnosis of GHD was made when two or more GH provocation tests yielded insufficient peak GH levels. All children were instructed to fast overnight for 8 to 12 hours before the test. Participants underwent serial blood sampling at 0, 30, 60, 90, and 120 minutes following an intravenous bolus injection of arginine and L-dopa, and the peak GH level was determined based on the results of the provocation tests. Serum GH levels were measured using an ELISA-based immunoassay. Given the well-established inter-assay variability of GH immunoassays and the tendency of ELISA platforms to report higher GH concentrations than modern chemiluminescent assays (24, 25), an assay-appropriate diagnostic cutoff of<10 ng/mL was applied in accordance with our institutional protocol. As a peak GH level >10 ng/mL indicates the absence of GHD, a cutoff value of 10 ng/mL was employed in the diagnostic evaluation (26). Based on the peak GH level, participants were categorized as having either GHD or ISS.

### 2.3 MRI image acquisition

MRI scans were acquired at the first hospital visit using a 3.0-T scanner (Philips Achieva, Best, Netherlands) equipped with a 16-channel phased array head coil and a magnetization-prepared rapid gradient echo sequence. All scans were performed without sedation, and an experienced pediatrician continuously monitored pulse oximetry to ensure stable cardiac and respiratory parameters throughout the procedure. The imaging protocol included whole-brain and standard pituitary MRI sequences, comprising sagittal and axial T1-weighted images. The parameters were: TE = 3.39 ms, TR = 2.10 ms, TI = 1 ms, field of view = 200 mm^2^, voxel size = 0.9 × 0.9 mm^2^, slice thickness = 1 mm, and slice number = 150.

### 2.4 Pituitary gland volume segmentation

Pituitary gland volume segmentation was performed using ITK-SNAP (version 3.6.0; University of Pennsylvania, PA, USA; http://www.itksnap.org), an open-source software offering manual and semi-automated segmentation functions. First, a DICOM image series was imported into ITK-SNAP. Segmentation of the pituitary gland was guided by anatomical landmarks, with the sphenoid sinus anteriorly, diaphragma sellae superiorly, cavernous sinuses laterally, and dorsum sellae posteriorly. Manual segmentation was performed on each sagittal slice using the paintbrush tool, carefully excluding Rathke’s cleft cyst if present. The volume of the segmented pituitary gland was automatically calculated in the “Volume and Statistics” module of ITK-SNAP, based on the total number of labeled voxels multiplied by the voxel volume.

### 2.5 Thalamic nuclei volume analysis

3D T1-weighted images were processed using the recon-all pipeline in FreeSurfer (version 7.2.0; Boston, MA, USA), which included surface reconstruction and cortical parcellation. Manual corrections, such as adding control points to adjust gray matter–white matter boundary errors, were applied and reprocessed as needed. Thalamic nuclei were segmented using an automated algorithm in FreeSurfer, based on a probabilistic atlas constructed from ex vivo MRI and histological data. The detailed methodology has been described previously (27). Segmentation accuracy was verified through visual inspection using Freeview. Total intracranial volume (tICV) was automatically extracted, and relative thalamic volume was calculated using the following formula:


$$\begin{array}{c} \text{Relative volume}\left(\%\right)\\ =\frac{\text{Absolute thalamic nuclei volume}}{\text{Total intracranial volume}}\times \;1,000 \end{array}$$

**Figure 1:**
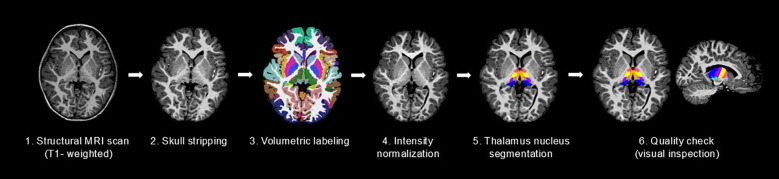
The pipeline for thalamic nuclei volume analysis. Overall flow chart of brain imaging preprocessing and segmentation of thalamus nucleus using FreeSurfer. Structural T1-weighted magnetic resonance images are shared by the recon-all. Thalamus nucleus segmentation produces volumes of the thalamus nucleus.

**Figure 2:**
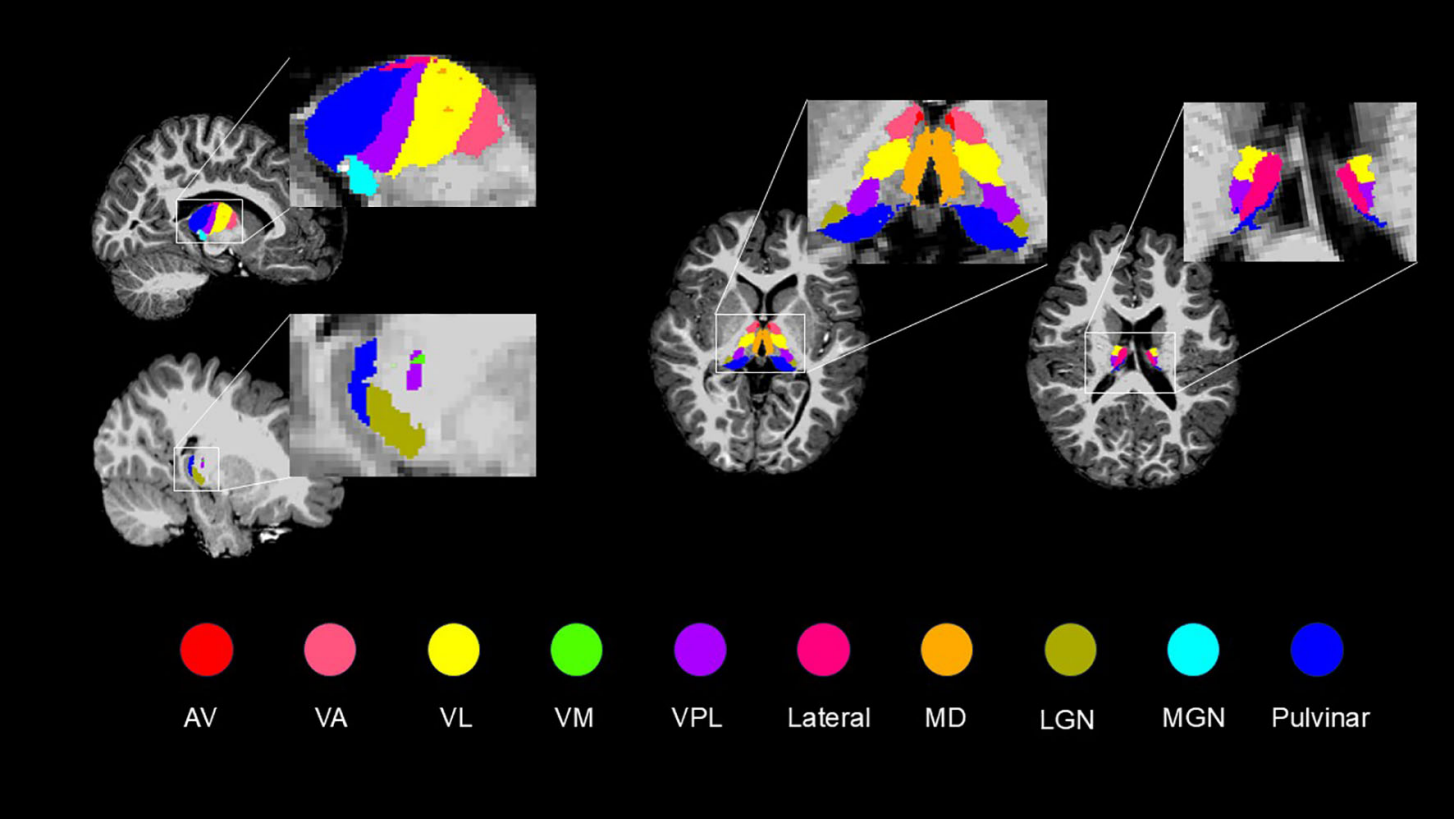
Grouped into ten categorise of thalamic nuclei. This figure illustrates ten categorized thalamic subnuclei used for thalamic nuclei analysis. Specifically, the VA group included the VA and VAmc nuclei. The VL group comprised the VLa and VLp nuclei. The Lateral group consisted of the LD and LP nuclei. The MD group included the MDm and MDi nuclei. The Pulvinar group encompassed the PuA, PuM, PuL, and PuI nuclei. VA, ventral anterior; VAmc, ventral anterior magnocellular; VL, ventral lateral; VLa, ventral lateral anterior; VLp, ventral lateral posterior; LD, laterodorsal; LP, lateral posterior; MD, mediodorsal; MDm, mediodorsal medial; MDi, mediodorsal intermediate; PuA, pulvinar anterior; PuM, pulvinar medial; PuL, pulvinar lateral; PuI, pulvinar inferior.

All images were independently reviewed by two researchers, and only those that passed quality control were included in the final analysis. The pipeline of data pre-processing and thalamic nuclei volume analyses is shown in **Figure 1**. The thalamic nuclei were grouped into 10 categories based on their anatomical location and functional similarity: Anteroventral (AV), Ventral anterior (VA), Ventral lateral (VL), Ventromedial (VM), Ventral posterolateral (VPL), Lateral, Mediodorsal (MD), Lateral geniculate (LGN), Medial geniculate (MGN), and Pulvinar. Specifically, the VA group included the VA and ventral anterior magnocellular (VAmc) nuclei. The VL group comprised the ventral lateral anterior (VLa) and ventral lateral posterior (VLp) nuclei. The Lateral group consisted of the laterodorsal (LD) and lateral posterior (LP) nuclei. The MD group included the mediodorsal medial (MDm) and mediodorsal intermediate (MDi) nuclei. The Pulvinar group encompassed the pulvinar anterior (PUA), pulvinar medial (PuM), pulvinar lateral (PuL), and pulvinar inferior (PuI) nuclei (**Figure 2**).

### 2.6 Statistical analysis

All statistical analyses were performed using IBM SPSS Statistics (version 27.0; SPSS Inc., Chicago, IL, USA) and R (version 4.4.2). For pituitary gland volume analyses, absolute volumes were analyzed using analysis of covariance (ANCOVA), with age, sex, and tICV. For thalamic nuclei analyses, volumes were normalized to tICV and multiplied by 1,000 to reduce variability related to overall brain size. Group comparisons of thalamic nuclei were subsequently performed using ANCOVA with age and sex as covariates. Sex differences between the two groups were analyzed using the chi-squared test. Student’s t-tests or Fisher’s exact tests were used to compare clinical variables, as appropriate. Effect sizes (Cohen’s *d*) were calculated for all measures including both significant and non-significant comparisons to provide a comprehensive description of group differences. *Post-hoc* power estimates based on the observed effect sizes were obtained using G*Power (version 3.1.9.7). For multiple comparisons, false discovery rate (FDR) correction was applied to analyses of the thalamic nuclei volumes. Statistical significance was defined as a two-tailed p-value less than 0.05, after FDR correction. Partial correlation coefficients (R values) were calculated to assess the associations between biochemical parameters and individual thalamic nuclei volumes, controlling for age and sex. A lateralization index (LI) was calculated to characterize the asymmetry of thalamic development using the following formula:


$$LI=\frac{Left-Right}{Left+Right}\times \;100$$

## 3 Results

### 3.1 Demographic and clinical characteristics

Demographic and endocrine clinical characteristics are shown in **Table 1**. Fifteen children (age range 4–6 years, mean 5.07 ± 0.80 years) with GHD and fifteen children (age range 4–6 years, mean 4.87 ± 0.83 years) with ISS were included in this study. Mean height was 101.65 ± 4.77 cm in GHD group, which was significantly shorter than the mean of 105.77 ± 3.37 cm for the ISS group (*p* = 0.017). Mean height SDS was also smaller in the GHD group (GHD: -2.10 ± 0.54 vs ISS: -1.21 ± 1.26, *p* = 0.031). In the GHD group, peak GH levels during both arginine and L-dopa stimulation tests were below 10ng/mL, whereas in the ISS group, peak levels exceeded 10ng/mL in at least one of the two tests (*p* = 0.026 for arginine and *p* = 0.017 for L-dopa). While IGF-1 and IGFBP-3 levels tended to be higher in the ISS group, these differences were not statistically significant.

**Table 1:** Demographics and clinical characteristics.

Clinical and biochemical characteristics	GHD group (mean ± SD)	ISS group (mean ± SD)	*p*
Characteristics			
Sex (male), n (%)	9 (60.0)	10 (66.7)	0.705
Age (years)	5.07 ± 0.80	4.87 ± 0.83	0.508
Height (cm)	101.65 ± 4.77	105.77 ± 3.37	0.017*
Height SDS	-2.10 ± 0.54	-1.21 ± 1.26	0.031*
Weight (kg)	15.96 ± 2.02	17.81 ± 1.96	0.021*
Weight SDS	-1.75 ± 1.05	-0.88 ± 1.11	0.042*
BMI (kg/m^2^)	15.40 ± 1.21	15.93 ± 1.28	0.275
BMI z-score	-0.42 ± 0.88	-0.06 ± 1.00	0.318
Bone age (years)	4.59 ± 0.92	5.01 ± 0.54	0.224
Biochemical parameters			
Peak GH-A (ng/mL)	5.46 ± 2.00	11.15 ± 6.21	0.026*
Peak GH-L (ng/mL)	5.90 ± 2.24	10.68 ± 6.68	0.017*
IGF-1 level (ng/mL)	122.80 ± 54.09	142.57 ± 61.32	0.405
IGF-1 SDS	-0.54 ± 0.69	-0.32 ± 0.75	0.453
IGFBP-3 level (ng/mL)	3612.93 ± 815.78	4124.80 ± 662.39	0.112
IGFBP-3 SDS	2.36 ± 1.27	3.46 ± 1.46	0.059
IGF-1/IGFBP-3 molar ratio	`0.12 ± 0.03	0.12 ± 0.04	0.831

The values are expressed as mean ± SD or n (%). *p< 0.05. GHD, growth hormone deficiency; ISS, idiopathic short stature; SD, standard deviation; SDS, standard deviation score; BMI, body mass index; Peak GH-A, Arginine; Peak GH-L, L-dopa; IGF-1, insulin-like growth factor-1; IGFBP-3, insulin-like growth factor-binding protein 3.Bold values with an asterisk (*) indicate statistical significance at p < 0.05.

### 3.2 Pituitary gland volume differences

**Figure 3:**
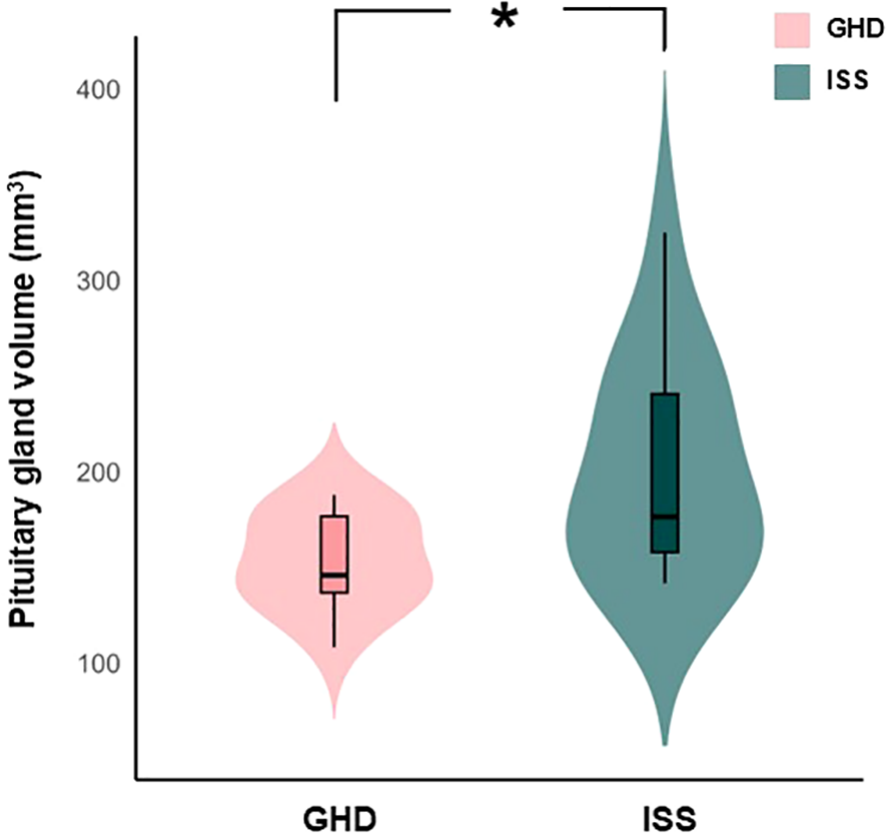
Differences in pituitary gland volume between children with GHD and ISS. This figure illustrates the comparison of pituitary gland volumes between children with GHD and ISS. Pituitary gland volumes were adjusted for age, sex, and tICV using analysis of covariance. Children with GHD exhibited significantly smaller pituitary gland volumes compared with those with ISS (*p* = 0.011). Error bars represent the distribution of values. *Statistically significant at p< 0.05. GHD, growth hormone deficiency; ISS, idiopathic short stature; tICV, total intracranial volume.

The mean volume of the pituitary gland was 153.650 ± 24.102 mm^3^ in the GHD group and 200.454 ± 54.130 mm^3^ in the ISS group. Children with GHD exhibited a significantly smaller pituitary gland volume compared with those with ISS, and this difference remained statistically significant after adjustment for age, sex, and tICV using ANCOVA (adjusted *p* = 0.011; **Table 2**, **Figure 3**). This group was associated with a large effect size (Cohen’s *d* = 1.117) and a high achieved *post-hoc* power (1-β = 0.840), indicating a robust between-group differences. Correlations between pituitary gland volume and biochemical parameters in the GHD group are summarized in **Supplementary Table 1**.

**Table 2:** Volumetric comparison of the pituitary gland between two groups.

Volume measures	All children					
	GHD group (mean ± SD)	ISS group (mean ± SD)	*p*	Adjusted *p^a^*	Cohen’s *d* (effect size)	*Post-hoc* power (1-β)
Pituitary gland volume (mm^3^)	153.650 ± 24.102	200.454 ± 54.130	0.005	0.011*	1.117	0.840

The values are expressed as mean ± SD. *p < 0.05. *^a^*Adjusted for age, sex, and tICV using analysis of covariance. Cohen’s *d* represents standardized effect size. *Post-hoc* power was calculated using a two-tailed test with α = 0.05 and equal group sizes. GHD, growth hormone deficiency; ISS, idiopathic short stature; SD, standard deviation; tICV, total intracranial volume.Bold values with an asterisk (*) indicate statistical significance at p < 0.05.

### 3.3 Comparison of volumes and LI of the thalamic nuclei

**Figure 4:**
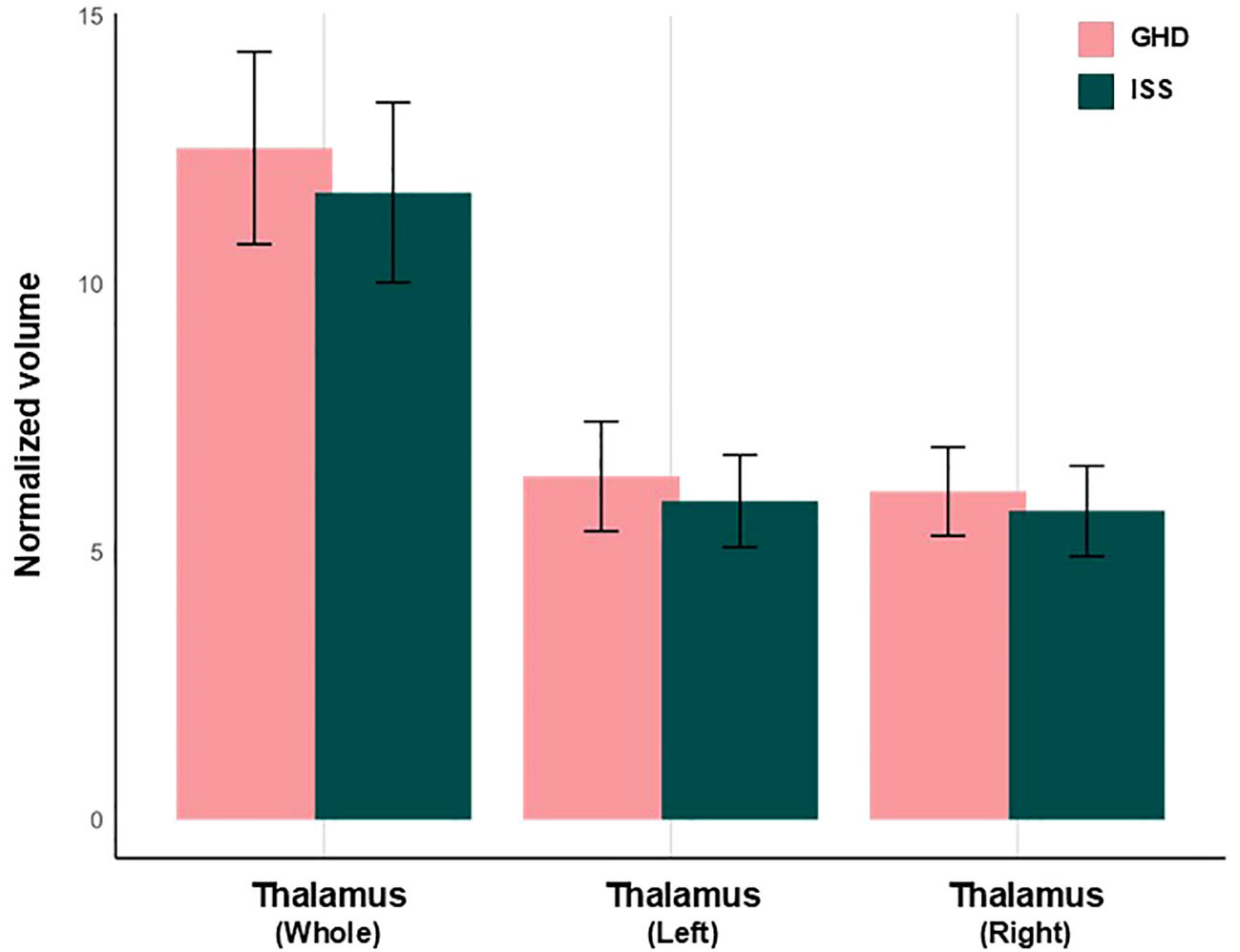
Differences in thalamus volume between children with GHD and children with ISS. Volume of the whole thalamus, left thalamus, and right thalamus are shown. All thalamic volumes were normalized to tICV and adjusted for age and sex. No significant group differenes were observed for the whole thalamus or for either hemisphere. GHD, growth hormone deficiency; ISS, idiopathic short stature; tICV, total intracranial volume.

**Figure 5:**
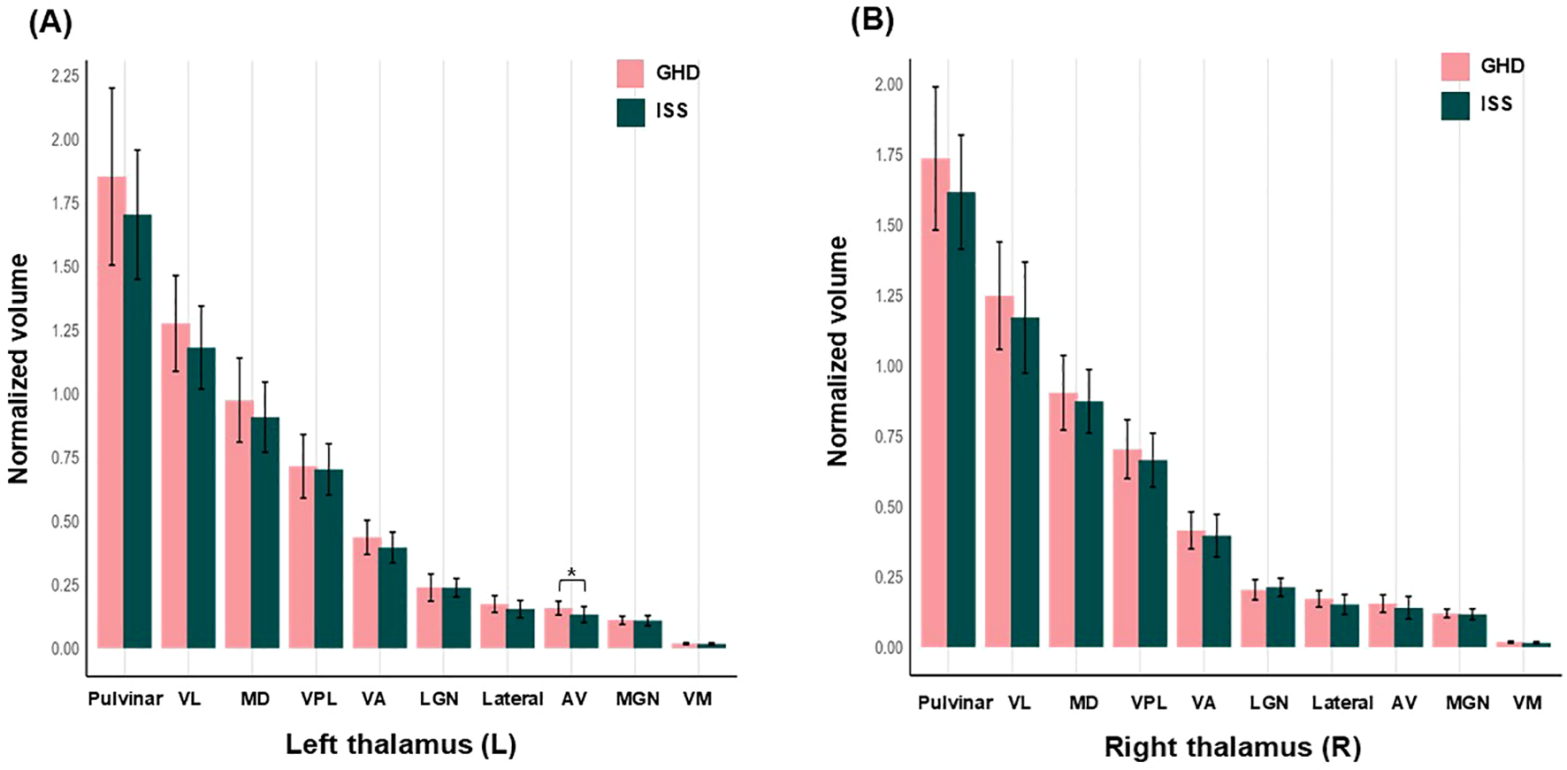
Thalamic nuclei volume differences between children with GHD and children with ISS. This figure shows the group differences in the volumes of left and right thalamic nuclei between children with GHD and ISS. All thalamic nuclei volumes were normalized to tICV and adjusted for age and sex. **(A)** In the left thalamus (L), children with GHD exhibited a significantly greater volume of the AV nucleus compared with children with ISS (*p* = 0.036). **(B)** In the right thalamus (R), no significant group differences were observed in any thalamic nucleus. *Statistically significant at *p* < 0.05. GHD, growth hormone deficiency; ISS, idiopathic short stature; tICV, total intracranial volume; VL, ventral lateral; MD, mediodorsal; VPL, ventral posterolateral; VA, ventral anterior; LGN, lateral geniculate; AV, anteroventral; MGN, medial geniculate; VM, ventromedial.

**Figure 6:**
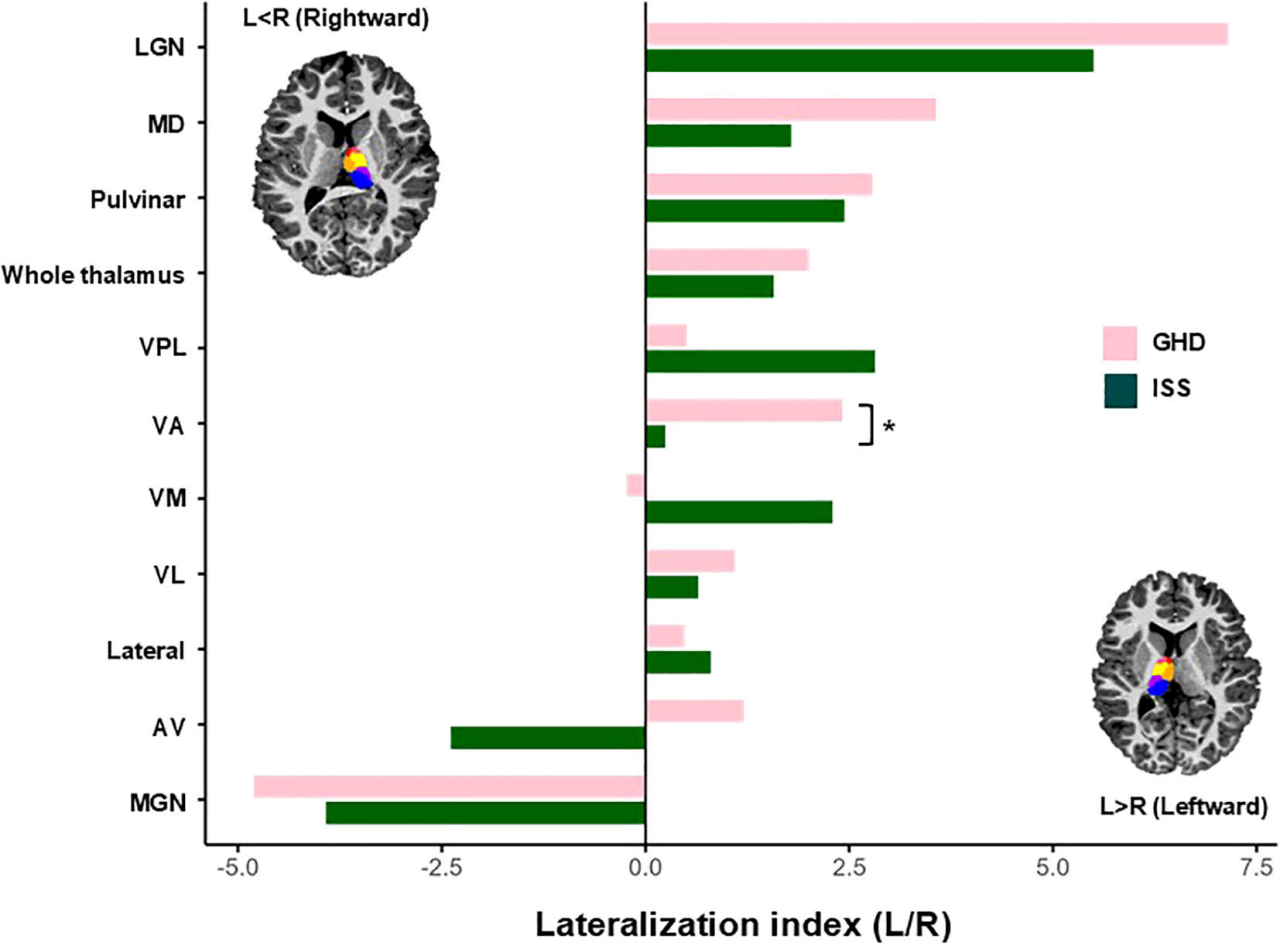
Mean lateralization index of the whole thalamus and thalamic nuclei. The LI of the whole thalamus and individual thalamic nuclei is shown for children with GHD and ISS. Positive LI values indicate leftward lateralization, whereas negative values indicate rightward lateralization. Children with GHD exhibited significantly more pronounced leftward lateralization in the VA nucleus compared with children with ISS (*p* = 0.023). Statistically significant at *p* < 0.05. LI, lateralization index; GHD, growth hormone deficiency; ISS, idiopathic short stature; VA, ventral anterior; LGN, lateral geniculate; MD, mediodorsal; VPL, ventral posterolateral; VM, ventromedial; VL, ventral lateral; AV, anteroventral; MGN, medial geniculate. *Statistically significant at p < 0.05.

**Table 3** shows the volumes of the whole thalamus and individual thalamic nucleus. Whole and bilateral thalamic volumes did not differ between the groups (**Figures 4**, **5**). However, a significant group difference was found exclusively in the left AV nucleus. The GHD group exhibited a significantly greater volume of the AV nucleus than the ISS group (0.159 ± 0.027 mm^3^ vs. 0.133 ± 0.031 mm^3^; *p* = 0.036), suggesting a localized structural alteration. The increased volume of the left AV nucleus in children with GHD was associated with a large effect size (Cohen’s *d* = 0.868), although the *post-hoc* power was moderate (1−β = 0.631), reflecting the modest sample size. To further investigate the relationship between thalamic structure, brain development, and functional characteristics, the LI of the thalamic nuclei were analyzed. The LI of the VA nucleus showed more pronounced leftward lateralization in children with GHD. This difference corresponded to a medium effect size (Cohen’s *d* = 0.746), with limited *post-hoc* power (1−β = 0.505). No other thalamic nuclei exhibited significant group differences (**Table 4**, **Figure 6**).

**Table 3:** Comparison of thalamic nuclei volume between two groups.

Volume measures	All children					
	GHD group (mean ± SD)	ISS group (mean ± SD)	*p*	Adjusted *p^a^*	Cohen’s *d* (effect size)	*Post-hoc* power (1-β)
Thalamus						
Whole thalamus	12.539 ± 1.795	11.708 ± 1.680	0.202	0.249	0.478	0.244
Left thalamus	6.410 ± 1.024	5.947 ± 0.862	0.191	0.231	0.490	0.254
Right thalamus	6.129 ± 0.827	5.762 ± 0.843	0.239	0.299	0.440	0.214
Thalamic nuclei						
Left						
AV	0.159 ± 0.027	0.133 ± 0.031	0.024	0.036*	0.868	0.631
VA	0.436 ± 0.067	0.396 ± 0.060	0.097	0.137	0.628	0.383
VL	1.276 ± 0.188	1.181 ± 0.163	0.152	0.197	0.537	0.295
VM	0.019 ± 0.004	0.018 ± 0.004	0.402	0.479	0.311	0.130
VPL	0.715 ± 0.125	0.703 ± 0.100	0.774	0.876	0.106	0.059
Lateral	0.174 ± 0.033	0.154 ± 0.034	0.112	0.155	0.599	0.354
MD	0.975 ± 0.165	0.908 ± 0.138	0.238	0.293	0.429	0.206
LGN	0.239 ± 0.053	0.238 ± 0.036	0.969	0.956	0.014	0.050
MGN	0.110 ± 0.016	0.109 ± 0.020	0.861	0.934	0.065	0.053
Pulvinar	1.853 ± 0.347	1.703 ± 0.254	0.189	0.197	0.492	0.256
Right						
AV	0.156 ± 0.031	0.141 ± 0.040	0.276	0.336	0.405	0.188
VA	0.416 ± 0.066	0.397 ± 0.076	0.171	0.591	0.267	0.109
VL	1.249 ± 0.191	1.171 ± 0.197	0.281	0.339	0.401	0.186
VM	0.019 ± 0.004	0.017 ± 0.004	0.111	0.149	0.600	0.355
VPL	0.704 ± 0.104	0.665 ± 0.096	0.294	0.340	0.390	0.178
Lateral	0.172 ± 0.029	0.152 ± 0.035	0.100	0.138	0.620	0.375
MD	0.904 ± 0.132	0.873 ± 0.113	0.504	0.589	0.247	0.100
LGN	0.205 ± 0.036	0.213 ± 0.032	0.491	0.520	0.255	0.104
MGN	0.121 ± 0.015	0.118 ± 0.019	0.603	0.796	0.192	0.080
Pulvinar	1.736 ± 0.254	1.616 ± 0.202	0.166	0.212	0.519	0.279

The values are expressed as mean ± SD. Bold values with an asterisk (*) indicate statistical significance at p < 0.05. ^*a*^: Adjusted for age, sex, and tICV using analysis of covariance. Cohen’s *d* represents standardized effect size. Post-hoc power was calculated using a two-tailed test with α = 0.05 and equal group sizes. Abbreviations: GHD, growth hormone deficiency; ISS, idiopathic short stature; SD, standard deviation; tICV, total intracranial volume; AV, anteroventral; VA, ventral anterior; VL, ventral lateral; VM, ventromedial; VPL, ventral posterolateral; MD, mediodorsal; LGN, lateral geniculate; MGN, medial geniculate.

**Table 4:** Comparison of the lateralization index of thalamic nuclei between two groups.

Nucleus	GHD group (mean ± SD)	ISS group (mean ± SD)	*p*	Adjusted *p^a^*	Cohen’s *d* (effect size)	*Post-hoc* power (1-β)
Thalamus	2.003 ± 3.885	1.572 ± 2.308	0.714	0.749	0.135	0.065
AV	1.208 ± 5.635	-2.389 ± 3.461	0.044	0.056	0.769	0.053
VA	2.413 ± 3.270	0.238 ± 2.516	0.051	0.023*	0.746	0.505
VL	1.098 ± 2.214	0.646 ± 2.033	0.564	0.534	0.213	0.087
VM	-0.237 ± 6.878	2.293 ± 4.769	0.251	0.192	0.428	0.205
VPL	0.502 ± 5.123	2.816 ± 3.268	0.151	0.132	0.539	0.297
Lateral	0.477 ± 3.920	0.800 ± 2.911	0.800	0.736	0.093	0.057
MD	3.565 ± 4.429	1.788 ± 3.934	0.311	0.255	0.424	0.202
LGN	7.155 ± 8.959	5.498 ± 6.595	0.693	0.569	0.211	0.086
MGN	-4.802 ± 4.514	-3.925 ± 3.585	0.524	0.560	0.215	0.088
Pulvinar	2.784 ± 8.559	2.442 ± 4.563	0.868	0.892	0.050	0.052

The values are expressed as mean ± SD. *p<0.05. ^*a:*^ Adjusted for age, sex, and tICV using analysis of covariance. Cohen’s *d* represents standardized effect size. *Post-hoc* power was calculated using a two-tailed test with α = 0.05 and equal group sizes. GHD, growth hormone deficiency; ISS, idiopathic short stature; SD, standard deviation; tICV, total intracranial volume; AV, anteroventral; VA, ventral anterior; VL, ventral lateral; VM, ventromedial; VPL, ventral posterolateral; MD, mediodorsal; LGN, lateral geniculate; MGN, medial geniculate.Bold values with an asterisk (*) indicate statistical significance at p < 0.05.

### 3.4 Correlations between the LI and biochemical parameters in children with GHD

**Figure 7:**
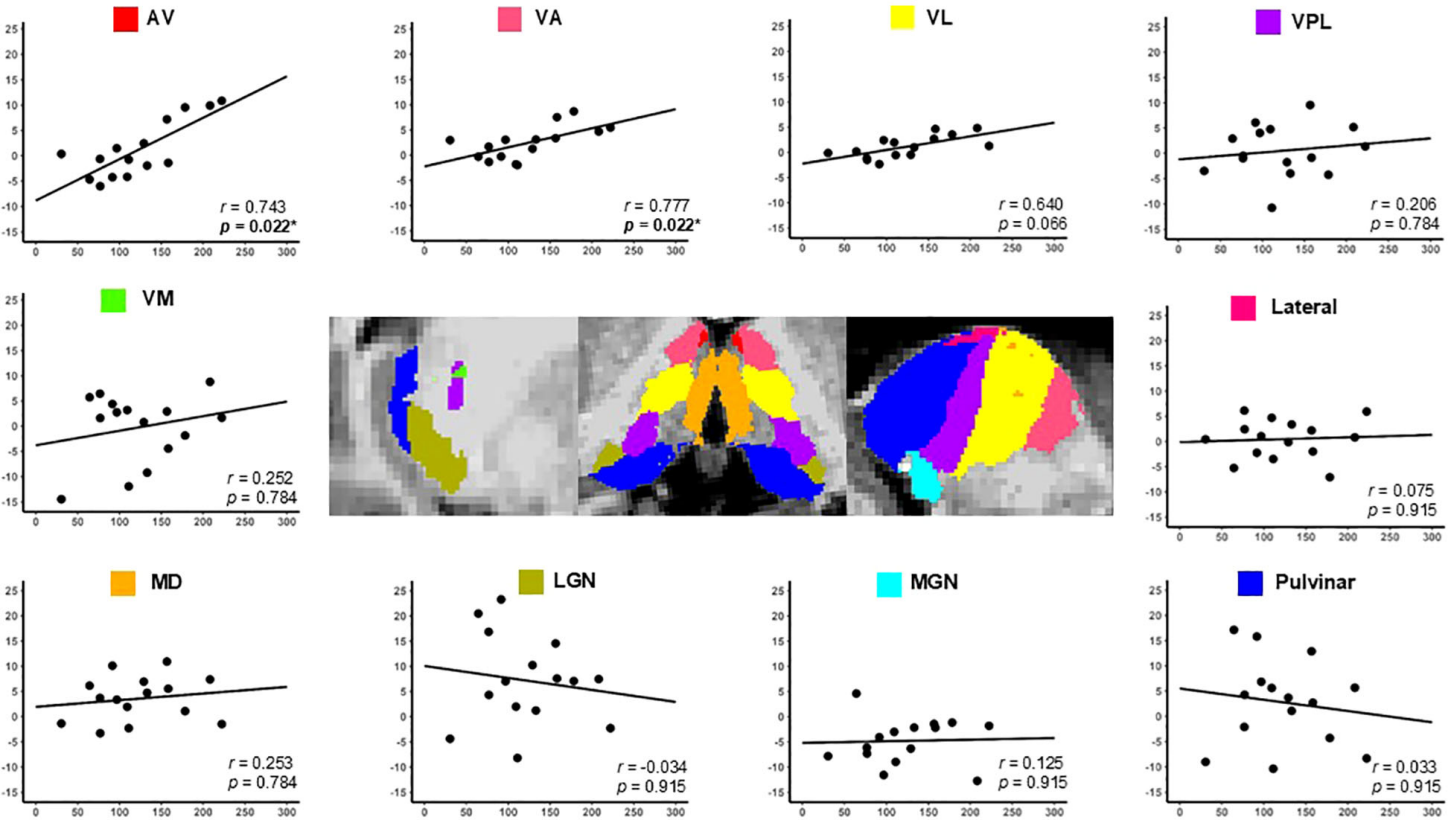
Correlation between the thalamic nucleus lateralization index and serum IGF-1 levels in the children with GHD. The y-axis represents the lateralization index of the thalamic nuclei, and the x-axis represents serum IGF-1 levels. Positive correlations were observed between serum IGF-1 levels and the lateralization indices in the AV and VA nuclei, with p-values of 0.022 for both nuclei. Statistical significance was set at *p* < 0.05. GHD, growth hormone deficiency; IGF-1, insulin-like growth factor-1; AV, anteroventral; VA, ventral anterior; VL, ventral lateral; VPL, ventral posterolateral; VM, ventromedial; MD, mediodorsal; LGN, lateral geniculate; MGN, medial geniculate. *Statistical significance was set at p < 0.05.

**Figure 8:**
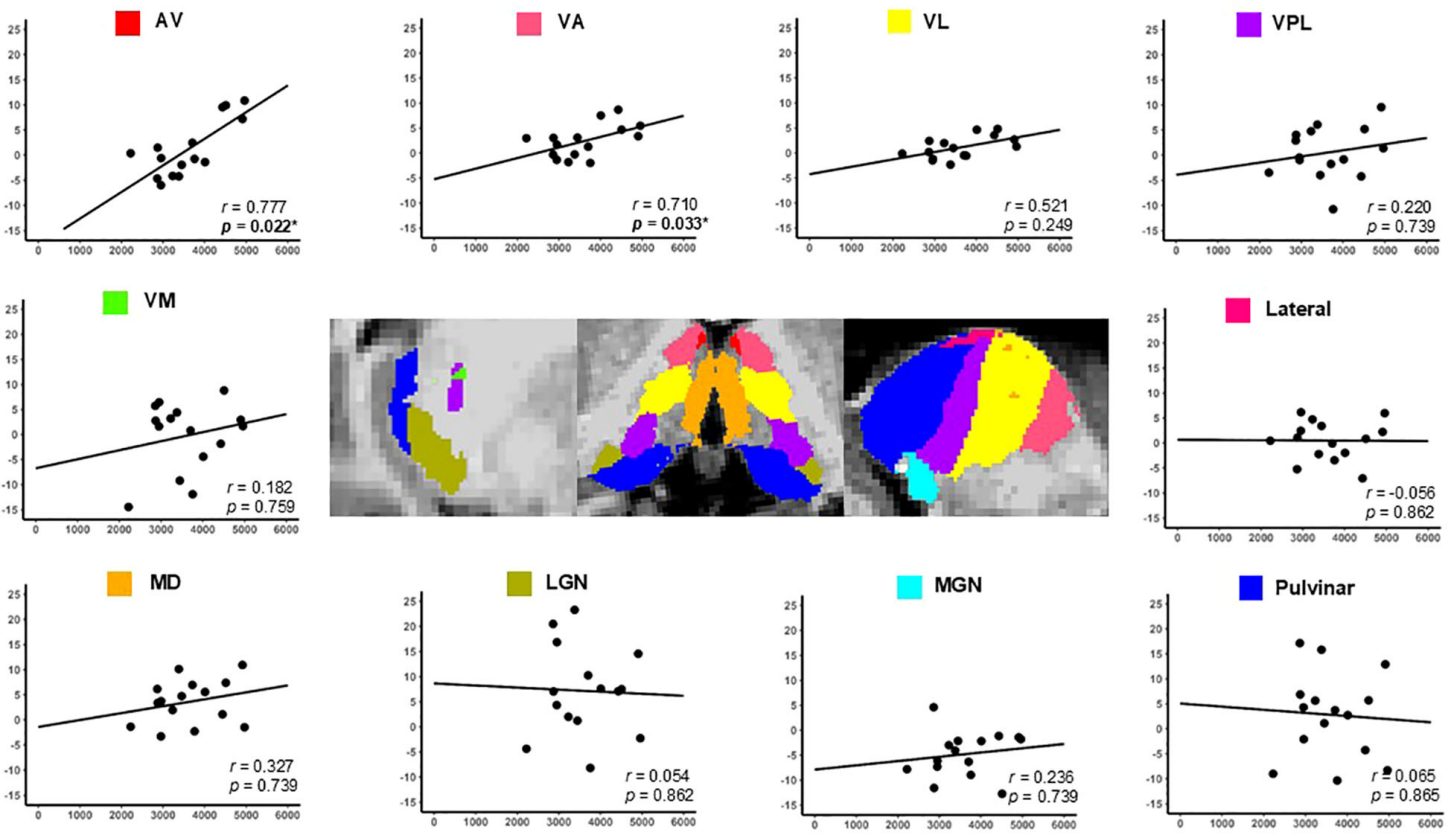
Correlation between the thalamic nucleus lateralization index and serum IGFBP-3 levels in the children with GHD. The y-axis represents the lateralization index of the thalamic nuclei, and the x-axis represents serum IGFBP-3 levels. Positive correlations were observed between IGFBP-3 levels and the lateralization indices in the AV and VA nuclei, with p-values of 0.022 and 0.033, respectively. *Statistical significance was set at *p* < 0.05. GHD, growth hormone deficiency; IGFBP-3, insulin-like growth factor-binding protein-3; AV, anteroventral; VA, ventral anterior; VL, ventral lateral; VPL, ventral posterolateral; VM, ventromedial; MD, mediodorsal; LGN, lateral geniculate; MGN, medial geniculate.

**Figures 7**, **8** illustrate the correlations between the LI of individual thalamic nuclei volumes and the serum IGF-1 and IGFBP-3 levels. Analysis revealed that higher IGF-1 and IGFBP-3 levels were significantly correlated with increased LI of the AV nucleus (FDR-corrected *p* = 0.022 for all). Additionally, the VA nucleus showed significant positive correlations with the IGF-1 (*p* = 0.022) and IGFBP-3 levels (*p* = 0.033). Consistent with these findings, IGF-1 SDS was also significantly associated with the VA nucleus (*p* = 0.033) and ventral lateral nuclei (*p* = 0.044). No other thalamic nuclei showed significant correlations following FDR-correction (**Table 5**). The correlations between thalamic nuclei volume and biochemical parameters are provided in **Supplementary Tables 2, 3**.

**Table 5:** Correlations between lateralization index of thalamic nucleus and biochemical parameters in children with GHD.

Biochemical parameters	Statistics	Thalamus	AV	VA	VL	VM	VPL	Lateral	MD	LGN	MGN	Pulvinar
IGF-1	r	0.234	0.743	0.777	0.640	0.252	0.206	0.075	0.253	-0.034	0.125	0.033
	*p* ^a^	0.442	0.004	0.002	0.018	0.406	0.499	0.809	0.404	0.913	0.684	0.915
	*FDR p*	0.784	0.022*	0.022*	0.066	0.784	0.784	0.915	0.784	0.915	0.915	0.915
IGF-1 SDS	r	0.167	0.656	0.759	0.699	0.154	0.195	0.160	0.111	-0.187	0.083	-0.020
	*p* ^a^	0.585	0.015	0.003	0.008	0.616	0.523	0.602	0.718	0.541	0.789	0.949
	*FDR p*	0.847	0.055	0.033*	0.044*	0.847	0.847	0.847	0.868	0.847	0.868	0.949
IGFBP-3	r	0.258	0.777	0.710	0.521	0.182	0.220	-0.056	0.327	0.054	0.236	0.065
	*p* ^a^	0.395	0.002	0.006	0.068	0.552	0.470	0.857	0.276	0.862	0.437	0.834
	*FDR p*	0.739	0.022*	0.033*	0.249	0.759	0.739	0.862	0.739	0.862	0.739	0.862
IGFBP-3 SDS	r	0.256	0.606	0.729	0.530	0.200	0.110	-0.216	0.310	0.130	0.323	0.092
	*p* ^a^	0.399	0.028	0.005	0.063	0.513	0.720	0.478	0.303	0.673	0.281	0.766
	*FDR p*	0.705	0.154	0.055	0.231	0.705	0.766	0.705	0.666	0.766	0.666	0.766
IGF-1/IGFBP-3 Molar ratio	r	0.214	0.550	0.720	0.666	0.270	0.133	0.109	0.217	-0.061	0.009	0.045
	*p* ^a^	0.482	0.051	0.005	0.013	0.372	0.665	0.724	0.477	0.843	0.976	0.883
	*FDR p*	0.884	0.187	0.055	0.072	0.884	0.971	0.971	0.884	0.971	0.976	0.971

*^a^*Controlling for age and sex as covariate in all children. *p<0.05. GHD, growth hormone deficiency; IGF-1, insulin-like growth factor-1; IGFBP-3, insulin-like growth factor-binding protein 3; SDS; standard deviation score; FDR, false discovery rate; AV, anteroventral; VA, ventral anterior; VL, ventral lateral; VM, ventromedial; VPL, ventral posterolateral; MD, mediodorsal; LGN, lateral geniculate; MGN, medial geniculate.Bold values with an asterisk (*) indicate statistical significance at p < 0.05.

## 4 Discussion

The aim of this study was to investigate morphological differences in the pituitary gland and thalamic nuclei between children with GHD and those with ISS, and to characterize developmental and lateralization patterns of the thalamus in children with GHD. The main findings of this study can be summarized as follows. Children with GHD exhibited a significantly smaller pituitary gland volume compared with those with ISS, even after adjustment for age, sex, and tICV. In addition, volume was increased in the left AV thalamic nucleus and more pronounced leftward lateralization of the VA nucleus were observed in children with GHD. Lastly, there were positive correlations between the LI of thalamic nuclei and serum IGF-1 and IGFBP-3 levels. Together, these findings suggest that GHD not only affects somatic growth but also induces structural and lateralization changes in subcortical brain regions, potentially reflecting altered neurodevelopmental processes mediated by GH/IGF axis activity.

### 4.1 MRI in the assessment of pituitary gland volume in children with GHD

Consistent with previous studies, our results demonstrate that children with GHD exhibited significantly reduced pituitary gland volumes compared to children with ISS. Importantly, this volumetric reduction remained statistically significant after adjustment for age, sex, and tICV. These findings suggest that pituitary gland volume represents a robust morphological feature associated with GHD during early childhood. Previous studies using conventional 2D MRI have similarly reported decreased pituitary morphology in children with GHD, although those studies often relied on geometric approximations (e.g., the ellipsoid formula), which may underestimate the true pituitary gland volume (28). By contrast, the present study employed semi-automated segmentation techniques applied to high-resolution 3D MRI, which allowed for more anatomically accurate and reproducible volume estimates. These findings support the clinical utility of 3D MRI pituitary gland volume in the evaluation of pediatric GHD and underscore the importance of considering tICV as a covariate when interpreting pituitary morphology during periods of rapid brain development.

### 4.2 Subnuclear and hemispheric thalamic alterations

Previous neuroimaging studies have consistently reported global brain morphometric abnormalities in children with GHD, including reductions in total brain volume, cortical surface area, and gray matter thickness (14). Region-specific atrophy has also been observed in structures such as the corpus callosum, hippocampus, and thalamus (4), implicating disruptions in interhemispheric integration, memory processing, and sensory integration. Diffusion tensor imaging studies further support the presence of microstructural white matter abnormalities, such as increased mean diffusivity in the corticospinal tract (29), which may contribute to altered motor function. Functional MRI studies have additionally demonstrated reduced structure–function coupling in the primary sensory and motor cortices among children with GHD (30), suggesting a decoupling between anatomical integrity and neural activation patterns. The present study extends previous research by identifying, for the first time, alterations in thalamic subnuclear morphology in children with GHD, a region that has received limited attention. In contrast to prior studies that have primarily focused on global volumetric reductions and cortical thinning, our results reveal a more nuanced pattern of subcortical remodeling. Notably, we identified an increased volume of the left AV thalamic nucleus in children with GHD. The AV nucleus is a key component of the Papez circuit (31), linking the hippocampus and cingulate cortex and contributing to memory-related processes. This focal volumetric alteration may reflect adaptive neurodevelopmental changes associated with disrupted GH signaling during early brain maturation.

In addition to volumetric alterations, children with GHD exhibited significantly more pronounced leftward lateralization of the VA nucleus compared to children with ISS. Hemispheric lateralization is a fundamental organizational principle of the developing brain, supporting functional specialization and neural efficiency (32, 33). This process follows a tightly regulated developmental trajectory during early childhood and is influenced by both genetic and hormonal factors. Structural asymmetries in subcortical regions, including the thalamus, have been associated with typical cognitive and motor development (34), and previous studies have reported pronounced leftward thalamic asymmetry during the preschool period (35). The VA nucleus occupies a strategic position within cortico–basal ganglia–thalamic loops and is extensively interconnected with the prefrontal and premotor cortices, as well as the basal ganglia (36–38). Through these connections, the VA nucleus plays a critical role in motor planning, initiation of voluntary movement, and higher-order cognitive processes such as executive control and response selection. The more pronounced leftward lateralization of the VA nucleus observed in children with GHD may therefore reflect asymmetric maturation of motor and cognitive circuits, potentially driven by region-specific sensitivity to GH/IGF signaling. Whether this pattern represents an adaptive compensatory mechanism or an early marker of altered hemispheric organization secondary to endocrine disruption remains to be clarified.

### 4.3 Association between thalamic asymmetry and GH–IGF axis activity

A key and novel contribution of the present study is the identification of significant positive correlations between thalamic asymmetry indices and biochemical markers of GH activity, specifically the serum IGF-1 and IGFBP-3 levels. Despite the absence of significant between-group differences in circulating IGF-1 and IGFBP-3 levels, the positive correlations observed within the children with GHD underscore an important distinction between group-level comparisons and individual variability. Prior neuroimaging studies in children with GHD have demonstrated that growth-related hormone levels, including IGF-1 and IGFBP-3, show significant associations with volumes of subcortical structures within the GHD group, even in the absence of significant between-group differences, supporting the notion that relative IGF signaling may be associated with subcortical neuroanatomy within affected populations (17). Whereas between-group analyses primarily reflect average hormonal status, within-group associations capture how inter-individual differences in residual GH/IGF axis activity may influence neurodevelopment under conditions of hormonal insufficiency. Thus, in children with GHD, even modest variability in IGF-1 and IGFBP-3 levels may represent biologically meaningful differences in residual IGF signaling, which could exert a disproportionate influence on the maturation and organization of subcortical structures, including hemispheric specialization. On this basis, thalamic lateralization in children with GHD may relate to disease severity along a continuous biological spectrum rather than a dichotomous diagnostic category. Greater alterations in thalamic LI may reflect more pronounced disruption of GH/IGF signaling, whereas relatively preserved IGF signaling may be associated with more typical hemispheric organization. By contrast, children with ISS exhibit largely intact GH/IGF axis function and may therefore lack a comparable biological gradient linking hormone levels to thalamic lateralization, potentially accounting for the absence of significant correlations within the ISS group.

IGF signaling plays a critical role in CNS development by promoting neural proliferation, maturation, survival, and growth (19). Consequently, even modest variations in circulating IGF-1 and IGFBP-3 levels may exert disproportionate effects on subcortical maturation during sensitive developmental windows. In the present study, this heightened sensitivity was reflected in the observed associations between IGF-related biomarkers and thalamic lateralization indices, suggesting that thalamic development may be especially responsive to relative differences in GH/IGF axis activity. In line with these observations, more pronounced leftward lateralization in the AV and VA thalamic nuclei was associated with higher circulating IGF-1 and IGFBP-3 levels. IGF-1 is known to exert neurotrophic effects by promoting synaptogenesis and axonal growth in regions such as the thalamus and hippocampus (10, 39), while IGFBP-3 modulates IGF-1 bioavailability by regulating its stability and tissue distribution (40). Given that the AV and VA nuclei are embedded within motor and cognitive relay circuits, including connections with the secondary motor cortex and cortico–basal ganglia–thalamic loops (41, 42), the restriction of these associations to specific subregions suggests preferential sensitivity of sensorimotor-related thalamic nuclei to GH/IGF signaling. Collectively, these findings indicate that inter-individual variability in GH/IGF activity may contribute to differences in thalamic lateralization through region-specific neurodevelopmental mechanisms.

## 5 Conclusion

This study provides novel evidence that GHD in children is associated with morphological alterations in subcortical brain structures, particularly the thalamic nuclei. We identified localized volumetric enlargement in the AV nucleus in children with GHD compared to those with ISS. Furthermore, altered hemispheric lateralization in the VA nucleus, and its correlation with the serum IGF-1 and IGFBP-3 levels suggests that there is a potential compensatory neurodevelopmental response to reduced GH signaling. The increasing leftward asymmetry observed in these nuclei under the influence of IGF signaling indicates that the GH/IGF axis may preferentially modulate neurodevelopmental pathways involved in sensory and motor processing. Collectively, these findings suggest that impaired GH signaling not only affects somatic growth but also contributes to adaptive neurodevelopmental remodeling in subcortical regions during early brain maturation.

## 6 Limitations

Several methodological limitations should be acknowledged. First, the sample size was relatively small, potentially limiting statistical power and the generalizability of the findings. Second, the absence of neurodevelopmental assessments precluded evaluation of the cognitive or behavioral implications of the observed structural differences. Third, the cross-sectional design precludes assessment of longitudinal changes in brain morphology during different stages of development. Future studies with larger cohorts, longitudinal follow-up, and multimodal imaging approaches are needed.

## Data Availability

The original contributions presented in the study are included in the article/**Supplementary Material**. Further inquiries can be directed to the corresponding author.
