# Supplementary material for: Morphological changes and lateralization of the thalamic nuclei in children with growth hormone deficiency
Source: Front Endocrinol (Lausanne). 2026 Mar 4;16:1678738. doi: 10.3389/fendo.2025.1678738 (PMC12995643; doi:10.3389/fendo.2025.1678738)
Supplement: Supplementary file 1 [file Table1.docx]

Supplementary Material

# Supplementary Tables

**Supplementary Table 1. Correlations between pituitary gland volume and biochemical parameters in the children with GHD**

| **Statistics** | **IGF-1** | **IGF-1 SDS** | **IGFBP-3** | **IGFBP-3 SDS** | **IGF-1/IGFBP-3 molar ratio** |
| --- | --- | --- | --- | --- | --- |
| r | 0.146 | 0.366 | -0.019 | 0.055 | 0.335 |
| *p ^a^* | 0.635 | 0.219 | 0.951 | 0.858 | 0.264 |
| *FDR p* | 0.951 | 0.660 | 0.951 | 0.951 | 0.660 |

***^a^***: Controlling for age and sex as covariate in all children. Abbreviations: GHD, growth hormone deficiency; IGF-1, insulin-like growth factor-1; SDS, standard deviation score; IGFBP-3, insulin-like growth factor-binding protein 3; tICV, total intracranial volume; FDR, false discovary rate

**Supplementary Table 2. Correlations between the volumes of thalamic nuclei and biochemical levels in children with GHD**

| **Volume measures** | **IGF-1** | | | **IGFBP-3** | | |
| --- | --- | --- | --- | --- | --- | --- |
|  | **r** | ***p*^a^** | ***FDR p*** | **r** | ***p*^a^** | ***FDR p*** |
| **Thalamus** |  |  |  |  |  |  |
| Whole thalamus | -0.113 | 0.714 | 0.904 | -0.084 | 0.786 | 0.993 |
| Left thalamus | -0.037 | 0.904 | 0.904 | 0.003 | 0.993 | 0.993 |
| Right thalamus | -0.200 | 0.513 | 0.904 | -0.185 | 0.544 | 0.993 |
| **Thalamic nuclei** |  |  |  |  |  |  |
| **Left** |  |  |  |  |  |  |
| AV | 0.059 | 0.848 | 0.976 | 0.135 | 0.660 | 0.996 |
| VA | -0.071 | 0.817 | 0.976 | -0.019 | 0.951 | 0.996 |
| VL | -0.047 | 0.878 | 0.976 | -0.036 | 0.908 | 0.996 |
| VM | -0.009 | 0.976 | 0.976 | 0.059 | 0.848 | 0.996 |
| VPL | -0.012 | 0.970 | 0.976 | 0.027 | 0.930 | 0.996 |
| Lateral | -0.078 | 0.799 | 0.976 | -0.037 | 0.903 | 0.996 |
| MD | 0.016 | 0.959 | 0.976 | 0.008 | 0.979 | 0.996 |
| LGN | -0.071 | 0.819 | 0.976 | -0.002 | 0.996 | 0.996 |
| MGN | -0.076 | 0.804 | 0.976 | 0.021 | 0.945 | 0.996 |
| Pulvinar | -0.033 | 0.913 | 0.976 | 0.013 | 0.965 | 0.996 |
| **Right** |  |  |  |  |  |  |
| AV | -0.307 | 0.308 | 0.751 | -0.255 | 0.401 | 0.844 |
| VA | -0.275 | 0.363 | 0.751 | -0.206 | 0.500 | 0.844 |
| VL | -0.221 | 0.468 | 0.751 | -0.181 | 0.553 | 0.844 |
| VM | -0.237 | 0.435 | 0.751 | -0.129 | 0.675 | 0.844 |
| VPL | -0.172 | 0.573 | 0.751 | -0.167 | 0.585 | 0.844 |
| Lateral | -0.139 | 0.652 | 0.751 | -0.038 | 0.902 | 0.902 |
| MD | -0.161 | 0.600 | 0.751 | -0.226 | 0.458 | 0.844 |
| LGN | -0.034 | 0.912 | 0.912 | -0.073 | 0.813 | 0.902 |
| MGN | -0.192 | 0.529 | 0.751 | -0.162 | 0.598 | 0.844 |
| Pulvinar | -0.128 | 0.676 | 0.751 | -0.137 | 0.655 | 0.844 |

***^a^***: Controlling for age and sex as covariate in all children. Abbreviations: GHD, growth hormone deficiency; IGF-1, insulin-like growth factor-1; IGFBP-3, insulin-like growth factor-binding protein 3; FDR, false discovery rate; AV, anteroventral; VA, ventral anterior; VL, ventral lateral; VM, ventromedial; VPL, ventral posterolateral; MD, mediodorsal; LGN, lateral geniculate; MGN, medial geniculate

**Supplementary Table 3. Correlations between the volumes of thalamic nuclei and biochemical SDS levels in children with GHD**

| **Volume measures** | **IGF-1 SDS** | | | **IGFBP-3 SDS** | | | **IGF-1/IGFBP-3**  **molar ratio** | | |
| --- | --- | --- | --- | --- | --- | --- | --- | --- | --- |
|  | **r** | ***p*^a^** | ***FDR p*** | **r** | ***p*^a^** | ***FDR p*** | **r** | ***p*^a^** | ***FDR p*** |
| **Thalamus** |  |  |  |  |  |  |  |  |  |
| Whole thalamus | -0.155 | 0.612 | 0.753 | -0.127 | 0.679 | 0.903 | -0.189 | 0.537 | 0.703 |
| Left thalamus | -0.097 | 0.753 | 0.753 | -0.037 | 0.903 | 0.903 | -0.117 | 0.703 | 0.703 |
| Right thalamus | -0.219 | 0.472 | 0.753 | -0.231 | 0.448 | 0.903 | -0.266 | 0.380 | 0.703 |
| **Thalamic nuclei** |  |  |  |  |  |  |  |  |  |
| **Left** |  |  |  |  |  |  |  |  |  |
| AV | -0.059 | 0.849 | 0.878 | 0.040 | 0.896 | 0.992 | -0.074 | 0.810 | 0.900 |
| VA | -0.152 | 0.620 | 0.878 | -0.044 | 0.887 | 0.992 | -0.154 | 0.615 | 0.900 |
| VL | -0.103 | 0.737 | 0.878 | -0.060 | 0.844 | 0.992 | -0.119 | 0.700 | 0.900 |
| VM | -0.091 | 0.769 | 0.878 | 0.026 | 0.934 | 0.992 | -0.123 | 0.690 | 0.900 |
| VPL | -0.057 | 0.852 | 0.878 | -0.015 | 0.962 | 0.992 | -0.105 | 0.733 | 0.900 |
| Lateral | -0.167 | 0.586 | 0.878 | -0.143 | 0.642 | 0.992 | -0.178 | 0.560 | 0.900 |
| MD | -0.047 | 0.878 | 0.878 | -0.033 | 0.915 | 0.992 | -0.009 | 0.977 | 0.977 |
| LGN | -0.120 | 0.695 | 0.878 | -0.003 | 0.992 | 0.992 | -0.140 | 0.649 | 0.900 |
| MGN | -0.091 | 0.768 | 0.878 | -0.018 | 0.953 | 0.992 | -0.205 | 0.502 | 0.900 |
| Pulvinar | -0.082 | 0.791 | 0.878 | -0.028 | 0.928 | 0.992 | -0.110 | 0.720 | 0.900 |
| **Right** |  |  |  |  |  |  |  |  |  |
| AV | -0.377 | 0.204 | 0.778 | -0.242 | 0.425 | 0.718 | -0.333 | 0.266 | 0.618 |
| VA | -0.354 | 0.235 | 0.778 | -0.232 | 0.445 | 0.718 | -0.347 | 0.246 | 0.618 |
| VL | -0.289 | 0.338 | 0.778 | -0.205 | 0.501 | 0.718 | -0.294 | 0.330 | 0.618 |
| VM | -0.231 | 0.447 | 0.778 | -0.193 | 0.528 | 0.718 | -0.365 | 0.219 | 0.618 |
| VPL | -0.199 | 0.514 | 0.778 | -0.141 | 0.646 | 0.718 | -0.209 | 0.494 | 0.618 |
| Lateral | -0.269 | 0.34 | 0.778 | -0.090 | 0.771 | 0.771 | -0.265 | 0.381 | 0.618 |
| MD | -0.136 | 0.658 | 0.778 | -0.258 | 0.395 | 0.718 | -0.153 | 0.619 | 0.688 |
| LGN | 0.087 | 0.778 | 0.778 | -0.158 | 0.607 | 0.718 | -0.066 | 0.831 | 0.831 |
| MGN | -0.172 | 0.575 | 0.778 | -0.267 | 0.378 | 0.718 | -0.258 | 0.395 | 0.618 |
| Pulvinar | -0.102 | 0.741 | 0.778 | -0.231 | 0.448 | 0.718 | -0.216 | 0.477 | 0.618 |

*^a^*: Controlling for age and sex as covariate in all children. Abbreviations: FDR, false discovery rate; IGF-1, insulin-like growth factor-1; SDS, standard deviation score; IGFBP-3, insulin-like growth factor-binding protein 3; AV, anteroventral; VA, ventral anterior; VL, ventral lateral; VM, ventromedial; VPL, ventral posterolateral; MD, mediodorsal; LGN, lateral geniculate; MGN, medial geniculate
